# Pandemic response strategies and threshold phenomena

**DOI:** 10.1016/j.gloepi.2023.100105

**Published:** 2023-04-07

**Authors:** Pieter Streicher, Alex Broadbent

**Affiliations:** a Department of Philosophy, University of Johannesburg, South Africa; b Department of Philosophy, Durham University, United Kingdom

**Keywords:** Covid-19, Elimination, Suppression, Mitigation, Lockdown, Pandemic preparedness, Pandemic planning

## Abstract

This paper critically evaluates the Suppression Threshold Strategy (STS) for controlling Covid-19 (C-19). STS asserts a “fundamental distinction” between suppression and mitigation strategies, reflected in very different outcomes in eventual mortality depending on whether reproductive number *R* is caused to fall below 1. We show that there is no real distinction based on any value of *R* which falls in any case from early on in an epidemic wave. We show that actual mortality outcomes lay on a continuum, correlating with suppression levels, but not exhibiting any step changes or threshold effects. We argue that an excessive focus on achieving suppression at all costs, driven by the erroneous notion that suppression is a threshold, led to a lack of information on how to trade off the effects of different specific interventions. This led many countries to continue with inappropriate intervention-packages even after it became clear that their initial goal was not going to be attained. Future pandemic planning must support the design of “Plan B", which may be quite different from “Plan A".

## Introduction

The objective of this paper is to critically evaluate the “Suppression Threshold Strategy” (STS) for pandemic response. Early in the Covid-19 (C-19) pandemic, a “fundamental” distinction between strategic objectives of suppression and mitigation was asserted in terms of aiming “to reduce reproduction number, R, below 1 (suppression) – and thus cause case numbers to decline – or to merely slow spread by reducing R, but not to below 1 [(mitigation)].” [1]. This distinction was considered fundamental because of the sharply divergent outcomes of achieving the threshold of suppression. Even “optimal mitigation policies… would still likely result in hundreds and thousands of deaths… and health systems being overwhelmed many times over.” The conclusion was that “for countries able to achieve it, this leaves suppression as the preferred policy option.” Suppression then became the focus of attention, with the most influential and well-resourced further efforts to model mitigation scenarios tending to serve the conclusion that suppression was much better, rather than to provide guidance to countries not able to achieve suppression.

The predictions of the particular report in which this distinction is explicitly drawn [1] have been widely (and perhaps excessively) discussed, and the discussion is often politically loaded. There have also been numerous attempts to evaluate whether “lockdowns worked” in various places or in general [2,3], and many efforts to assess their costs [3,4]. There have also been efforts to assess the economic and health consequences of lockdowns, and criticisms of their use in contexts of poverty and overcrowding [5]. However, none of these discussions addresses the two fundamental elements of STS that we seek to address in this paper.

The first of these elements is that a strategically important or “fundamental” distinction exists between seeking to bring *R*_*t*_ below 1 and seeking to reduce it but not to a level below 1. (*R*_*t*_ is the reproduction number *R* some specified time *t*, and this clarification is important for reasons explained below.) The second element is the assumption that there is no need to articulate a distinct strategy for the situation in which suppression is not achieved. This assumption is not stated but it is implicit in the silence on what countries should do if they cannot achieve suppression. It is also implicit in the greater priority given to estimating disease burden as opposed to burdens imposed by disease control measures, for reasons explained below.

In this paper, we develop two corresponding criticisms of the Suppression Threshold Strategy. First, we show that the distinction between mitigation and suppression is not strategically fundamental, because outcomes lie on a continuum and do not, as the Strategy supposes, rapidly and widely diverge dependent on whether (or, more properly, when) *R*_*t*_ < 1 is reached. Second, we emphasise that the Suppression Threshold Strategy is silent on what to do if the suppression threshold is missed. The scenario in which countries locked down and *R*_*t*_ remained above 1 for an extended period was rare in the first wave, often due in part to the lateness of the measures, as discussed below. However, it did arise in some places, for example South Africa and Melbourne Australia (2nd and 6th lockdowns) [6,7]. There was no Plan B for these places, nor for places which did not or could not persist with hard and early lockdowns and/or did not achieve suppression until vaccination in subsequent waves, which ultimately included a large number of countries. In this strategic gap, the widespread assumption was that a redoubling or extending of efforts was the appropriate response to missing the putative suppression threshold. We show that, in some situations, the correct response is actually to *lift* the costliest measures when it becomes clear that a threshold will not be attained. This illustrates the importance of planning for situations in which a target threshold is missed, or turns out not to be a real threshold at all.

## Absolute values of *Rt* are not appropriate strategic objectives

*R*_*t*_ is the average number of people that each infected person infects at time *t*. The number of infections is growing when *R*_*t*_ > 1 and shrinking when *R*_*t*_ < 1. In the abstract, it may seem almost a matter of definition that measures designed to cause *R*_*t*_ below 1 are seeking to *suppress* the epidemic wave while those seeking to reduce R_t_ but not necessarily below 1 are seeking merely to *mitigate* the epidemic wave it. This is the basis of the aforementioned “fundamental” difference in strategies identified which formed a basis for early response measures in many places.

There are two reasons why this abstract and seemingly simple analysis is incorrect. The first is that it is meaningless without a timeframe. *R*_*t*_ is in decline from an early point in an epidemic wave and eventually falls below 1 in all scenarios (no matter what the mortality), since infections cannot grow to infinity. Thus a timeframe is needed in order to give sense to this way of drawing the distinction between suppression and mitigation. This is to say: we must specify how long we expect it to take for an intervention to work.

However, deciding on a timeframe presents difficulties. We do not expect an intervention to have a measurable outcome in case count on the very day it is introduced, because there is an incubation period, about which there may be considerable uncertainty. There are further lags until ascertainment, hospitalisation, and death, all of which may also be uncertain. There is also likely to be some fuzziness around the exact start point because people may begin adjusting behaviour before a lockdown (or, in not a few cases, engage in an intensified programme of social engagements). Moreover, these may be sources of variation in the underlying reality, as well as epistemic uncertainties: people may change their behaviour at different times, the disease may progress at different rates in different people, etc. In short, the expectation timeframe for evaluating the effectiveness of an intervention on any of the outcomes of interest, including *R*_*t*_, is a complex matter of judgment under uncertainty. This undermines any suggestion that it can form the basis of a “fundamental” strategic distinction.

Second, even if we could pinpoint a timeframe for *R*_*t*_ to fall below 1 with some degree of objectivity, *R*_*t*_ < 1 is still not a fundamental strategic indicator when taken out of context. A short, sharp wave may result in *R*_*t*_ falling below 1 sooner than a longer, flatter wave, even if many more people die in the short, sharp wave. The overriding measure of success is eventual attack rate (AR). If we focus on *R*_*t*_ out of context then we get many paradoxical results. For example, the exact same package of measures (e.g.: a “hard lockdown”) could count as mitigation if implemented early, because it might reduce *R*_*t*_ but not bring it below 1 in the specified timeframe. But the same package might count as suppression if implemented later when *R*_*t*_ was about to fall below 1 anyway [8]. Then the contribution made by the intervention package might be just enough to bring *R*_*t*_ below 1 within the timeframe (when otherwise it might have been a day later, say) and thus the interventions succeeded in suppression according to this way of viewing the matter. However, this is quite paradoxical. Not only are they the exact same package of measures, but in the scenario where they are introduced early (and thus count as mitigation by this definition) they save many more lives.

Therefore, if this way of drawing the distinction between suppression and mitigation is insisted upon, then we reach the conclusion that mitigation can sometimes save many more lives than suppression. We also must conclude that the very same package of measures can be either suppression or mitigation depending when it is introduced. Obviously these consequences contradict the idea that there is a fundamental strategic distinction in play, as well as contradicting many of the claims made about it. Defining strategies in terms of an absolute value of *R*_*t*_ taken out of context does not help clarify the conceptual and strategic situation.

One might propose an alternative threshold: the healthcare capacity of the nation (e.g.: number of critical care beds). Certainly, it was an important objective not to exceed healthcare systems' capacity. However, this is represented by some absolute value of important variables such as number of critical care beds and not by *R*_*t*_. It would naturally be called “healthcare capacity threshold” or something similar, and not “suppression threshold”. Moreover, even healthcare capacity cannot be treated as a fixed number. Healthcare systems adapted to the circumstances [9], as did individuals. Regardless, our contention in this paper is that a strategically significant distinction between suppression and mitigation cannot be drawn in terms of an absolute value of *R*_*t*_.

Reproductive number is a useful measure: it can convey very important information for disease control. However, it is an average measure responding to a number of different factors. There may be high transmission rates in some sub-populations and lower rates in others, and fluctuations in the population-level average *R*_*t*_ may not tell much about these, as has long been emphasised [10]. Treating some value of *R*_*t*_ as an important strategic target may make sense within a specific disease control effort embedded in a network of background assumptions and conditions. However, treating any value of *R*_*t*_, whether 1 or otherwise, as marking a fundamental distinction between epidemic response strategies is paradoxical in theory and misleading in practice. Put crudely, *R*_*t*_ < 1 is not a magic number.

## Clarifying the distinction between suppression and mitigation

Setting aside *R*_*t*_, a useful distinction between suppression and mitigation remains in terms of whether the focus is on reducing transmission in a whole population (suppression), or reducing mortality in specific groups (mitigation, focused protection, etc.). Here are our preferred definitions in terms of attack rates (AR).

SUPPRESSION STRATEGY [CLARIFIED] (SSc): Seeking to minimise population-level AR for a fixed period (e.g. until widespread vaccination).

MITIGATION STRATEGY [CLARIFIED] (MSc): Seeking to minimise AR in high risk settings such as care homes, hospitals, shelters, and high density neighbourhoods, through case isolation and targeted quarantine measures.

These strategies obviously share the larger goal of bringing eventual mortality down, but they are different ways to “win the war”. SSc seeks to minimise transmission within the population and thus reduce mortality (to whatever extent is decided in that instance). MSc seeks to minimise transmission among those most at risk and thus reduce mortality (again to whatever extent is targeted in that instance). These different ways to reduce overall mortality correspond to a distinction well-known in public health between targeting high-risk groups and targeting whole populations [11].

However, even on our preferred definitions, the distinction between suppression and mitigation is not fundamental. They are not mutually exclusive alternatives. Both approaches are likely to form part of any reasonable strategic response: special steps will be taken to protect vulnerable people even in a population-wide lockdown; and even in an approach focusing on vulnerable groups, some population-level measures will be introduced (e.g. restrictions on large gatherings, hand-washing campaigns). Both approaches also depend for their effectiveness on a degree of herd immunity building up. The difference is in the focus or balance between these approaches, the degree of population-wide restrictions, and perhaps even to some extent the messaging.

For our purposes, we set aside mitigation strategies (those targeting vulnerable groups) for the remainder of the paper, focusing instead on efforts aiming to reduce transmission in entire populations.

## Suppression is a matter of degree

We have made theoretical arguments that suppression is not a sharply defined category. If they are correct then we should expect to see suppression outcomes fall on a continuum. We now show that this is what actually transpired in the C-19 pandemic.

SSc was widely implemented, often through stringent population-level control measures (“lockdowns”), and its outcomes fell on a continuum. We considered the top 46 countries by GDP per capita as they were more likely to have the means for accurate surveillance. The pandemic was divided into two equal periods: March 2020 until end June 2021 (16 months), during which all countries applied restrictions to minimise attack rates prior to vaccination rollout; and July 2021 until 1 November 2022 (16 months), during which all countries started lifting restrictions allowing the virus to spread. Although different countries vaccinated at different times, our concern here is to gauge the level of suppression (on our definition, i.e. the extent to which population attack rates were minimised) while most countries in the group were still aiming at this. Hence the cut-off period of 1 July 2021 was chosen as being just before the first countries started to relax all restrictions.

We then calculated a *suppression proportion* (SP) of the pandemic during this interval as follows, where C_SUP_ is the cumulative case count on 1 July 2021 (i.e. cases during the suppression period) and C_TOT_ is the cumulative cases on 1 November 2022 (i.e. total cases).

$$\mathrm{SP}=\frac{\left(C_{\mathrm{TOT}}-2C_{\mathrm{SUP}}\right)}{C_{\mathrm{TOT}}}$$

SP of 0% means that half of the total cases happened in the first 16 month period and half of cases happened in the second 16 month period. In that instance, there is no difference between the period in which suppression was being undertaken and the period in which it was not. The proportion of cases that have been suppressed is 0% if there are no other factors in play. SP of 100% means that 100% of cases happened in the second 16 months, i.e. outside of the suppression period, and thus the proportion of the total that was suppressed was 100%, if no other factors are in play.

Naturally many other factors are in play, however. The timing of waves and the emergence of new variants, for example, could result in differences even in a hypothetical situation in which the exact same interventions remained in place across the entire 32 months. Nonetheless, our goal is not to draw a causal inference but to establish a *correlation* between timing of cases and eventual mortality. We are seeking to establish whether there is a correlation between the extent to which cases were “postponed” until after vaccines were available and eventual mortality. Using SP for this purpose does not imply any causal assumptions.

**Fig. 1:**
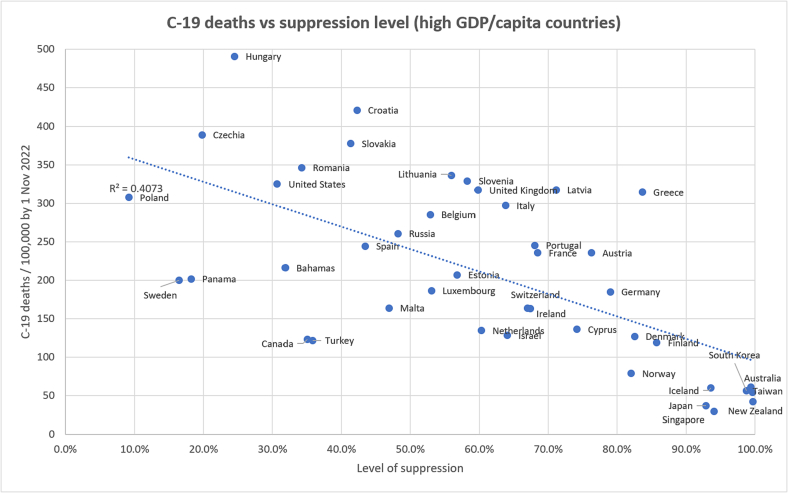
Covid-19 deaths by population as on 1 November 2022 plotted against levels of suppression achieved prior to 1 July 2021 in 46 high GDP/capita countries (excluding Saudi Arabia, Qatar and Brunei due to implausibly low case counts).

In Fig. 1, 100% on the X-axis indicates a maximal level of suppression as all C-19 cases happened during the 2nd period, after 1 July 2021, when restrictions were relaxed. 0% on the X-axis indicates equal cases in the 1st period compared to the 2nd period i.e., there was little if any suppression achieved during the 1st period. Total C-19 deaths by population for the whole period (up to 1 November 2022) was plotted on the Y-axis. The aim here was to see if there was any association between higher suppression levels and lower ultimate C-19 deaths, i.e. are there signs that delaying infections might have had a benefit in terms of reduced C-19 fatalities. Case and deaths data by population were obtained from the 91-DIVOC data visualisation tool using data from Johns Hopkins University CSSE [12].

A reduction in ultimate C-19 deaths was associated with an increase in suppression levels (Correlation coefficient = 0.4073). This was as expected and suggests that delaying infections until after vaccination had a beneficial effect in terms of ultimate C-19 deaths. (There will, as we mentioned already, be other factors in play. Notably, the emergence of a milder variant, Omicron, also helped in this regard, i.e. delaying infections until the emergence of Omicron also reduced ultimate C-19 deaths.)

There is a wide range of ultimate outcomes (200–491C-19 deaths/100 k) for those countries with low levels of suppression (0–30%) indicating that there must be other confounding factors that affected ultimate C-19 deaths. In contrast, all countries with very high levels of suppression (>90% for cases) ended with very low C-19 mortality levels (<61C-19 deaths/100 k). This category includes the countries that succeeded in eliminating the virus for long periods prior to vaccination. This is to be expected since if elimination occurs then the other factors resulting in varying outcomes cease to be relevant.

Below find a summary of suppression level and ultimate C-19 deaths:

Suppression level 0–30%, 200-491C-19 deaths/100 k.

Suppression level 30–50%, 130-421C-19 deaths/100 k.

Suppression level 50–70%, 130-340C-19 deaths/100 k.

Suppression level 70–90%, 80-320C-19 deaths/100 k.

Suppression level 90–100%, 29-61C-19 deaths/100 k.

Considering Fig. 1, it is apparent that suppression is not a threshold phenomenon as the outcome (mortality) of the vast majority of high GDP per capita countries lies on a continuum depending on the level of suppression achieved. We only see consistently low C-19 deaths when suppression levels exceeded 90% for a handful of countries, mostly island nations, that pursued and achieved elimination.

As others have pointed out, elimination is a very different goal to suppression, which falls within a broader category of control measures that includes mitigation [13]. The data supports the common-sense notion that elimination is a threshold phenomenon (since if there are no cases except those that are detected and isolated, there can be no transmission). This is a true threshold and is not one that is drawn in relation to a value or *R*_*t*_ but in relation to case counts. Achieving and sustaining a low case count for an extended period of time has a major effect on eventual AR. However, that there is no “suppression threshold” at any higher level than elimination. This is important because elimination was widely considered an unachievable objective.

Our analysis confirms the widespread view that minimising attack rates prior to vaccination improved eventual mortality. However, there is no magic threshold of suppression that yields a step change in mortality. Outcomes lie on a continuum: the more suppression was achieved, the better the eventual outcomes. Thus no basis remains for thinking there is a fundamental strategic distinction between suppression and mitigation strategies either in theory or practice.

## Plan A vs. Plan B

We now turn to our second criticism of STS, namely, its silence on what to do if a suppression effort fails to tip outcomes over a threshold. This situation may arise because the threshold turns out to be an illusion, and it becomes obvious that outcomes are falling on a continuum (as was the case for C-19 suppression). Or it may arise because the threshold, while real, is unattainable (as elimination might be in many places).

The justification for a package aiming at a threshold is logically quite different from the justification for a package aiming to move outcomes on continuum. If one can achieve a threshold then one enjoys the entire benefit of that threshold. Huge efforts may justify relatively small contributions to reducing transmission, because reaching the threshold makes such a huge difference to eventual mortality. However, a different intervention package, in which the costs and benefits of individual measures are considered, might be appropriate if there is no threshold to be crossed.

For example, if a country has achieved near-elimination, then closing schools might be a desirable measure even if children are at relatively low-risk and schools do not contribute majorly to transmission. (We are using school closures as a plausible [14,15], but strictly hypothetical, example.) However, if school closures did not tip the balance in favour of a dramatically improved outcome, then their modest direct contribution to reducing transmission might not be sufficient to outweigh their wider human costs.

This principle can have striking consequences. It could mean that restrictions on social contact should be relaxed even if cases subsequently rise—or even *while cases are still rising (as actually happened in South Africa)*. Once it is clear that a threshold cannot be reached, it becomes necessary to assess the additional benefit against the additional human cost of including each measure in the overall intervention package. The result might be that the additional benefit of including a measure in an intervention-package is rather modest, while its cost is rather large, and thus it is not worth including. That modest benefit would have been worthwhile if it had enabled us to cross a threshold and enjoy a very large benefit. But if the threshold has been missed or was an illusion, then the situation changes completely, and we are back to weighing up costs and benefits.

To continue with the school closure example, suppose schools are closed initially, but it later becomes clear that a threshold of elimination is not achieved (or that suppression outcomes are lying on a continuum). Previously school closures were justified despite their high cost because including them enabled the whole intervention-package to cross a threshold that resulted in far lower mortality. However, this no longer being the case, the justification for incurring these costs no longer exists. We are obliged to consider whether the benefits of closing schools in terms of eventual mortality outweigh the harms of doing so. Since the harms are considerable while the benefits may be smaller relative to some other measures (hypothetically), the school closures may become unjustified. Then the appropriate response may be to re-open schools, *even if cases are still rising or start to rise subsequently.*

This may feel quite paradoxical. “Common sense” may suggest that minimising transmission remains the appropriate goal even if we are not getting the results we hoped for. Imagine braking hard to prevent a collision. You will continue braking hard even when it is obvious that collision is inevitable, because that will reduce the impact.

However, common sense is a poor guide to public health strategy, and can yield conflicting results. Imagine you are running for a train, and you realise you will just fail to reach the doors in time. You could leap for the train as it passes, or keep running towards your destination as hard as you can. However, you would probably instead wait for another train. It must be acknowledged that epidemic suppression is not quite like this, because there is *some* benefit to retaining costly measures. Nonetheless, the step change that was hoped for can be missed (like a train), and it is in the nature of a threshold that missing it makes a big difference. This implies that you will need to reconsider, and it is likely you may need to change course.

Where threshold phenomena in epidemic response strategies are concerned, Plan B may be quite different to Plan A. Future pandemic strategies need to appreciate this point and plan for scenarios in which the threshold is missed, for whatever reason. In the C-19 pandemic, evaluation of the comparative benefit of different packages was initially discouraged because the consequences of failing to hit the putative suppression threshold were depicted as dire. The impression was created that trading off costs and benefits was a waste of valuable time and that all strategies short of maximal suppression would have disastrous outcomes. In effect, all “mitigation” strategies were bundled together along with doing nothing at all [16]. However, it was quite foreseeable that many nations would never achieve the requisite 75% reduction in social distancing [5] and that some suppression efforts would fail. The lack of guidance for Plan B was unfortunate and needs to be addressed in planning for future pandemics.

## Conclusion

The introduction of the idea of a suppression threshold in early 2020 galvanised the world into action, but unfortunately both the idea and the strategy it gave rise to were mistaken. The threshold of *R*_*t*_ < 1 was theoretically unsound, and turned out not to be a strategically significant threshold in the real world. Suppression is properly understood as the minimisation of population-level attack rates during a specified time period, e.g. until vaccination. Suppression outcomes lie on a continuum. We illustrate this with reference to C-19 outcomes from 46 high-GDP countries. Elimination, on the other hand, is a true threshold: reaching it causes a step change in outcomes. However, elimination represents an entirely different strategy from suppression [13], and it is a threshold that few countries could hope to obtain.

Our second criticism of STS is that it failed to provide, and discouraged providing, information on the relative merits of different control measures. This had negative consequences both for countries that did not meet the putative threshold, and for countries that did, given that the threshold was not real. If there is no threshold to be met, then the benefit of meeting that non-existent threshold cannot determine an optimal response, and cannot be used to justify the implementation of any measures. The difficult business of assessing their respective merits and setting these against their costs must be attempted. Repeated assertions that an “out of control” epidemic will result if *R*_*t*_ rises above 1 are conceptually ill-founded and proved false in the C-19 pandemic, as we have shown.

Modelling efforts simply did not focus on providing information about the relative merits of different mitigation scenarios. Perhaps this was because modellers were worried about appearing to endorse a strategic objective they strongly believed would be disastrous. Regardless of the reasons, this lacuna in the available intelligence led to a widespread inference that the best thing to do, in the scenario where a threshold is not crossed, is to keep on trying. However, this is not the case. Plan A and Plan B are often not the same when it comes to controlling infectious disease. Simplification resulted in a number of problematic messages during the pandemic, which were often unhelpful and sometimes even dangerous. Other examples include “Follow the science” and “Flatten the curve” [17]. Pandemic planning needs to find a way to overcome political and messaging issues. In this instance, it must articulate a clear Plan B for contexts where, for whatever reason, exceeding a set threshold is not attainable.

We therefore conclude by making two recommendations.

1. Future strategies should not regard suppression as a threshold, but as a matter of degree, with mortality outcomes lying on a continuum. Elimination is the only threshold for diseases relevantly similar to C-19.
2. Future strategies should provide for a range of scenarios, including those in which interventions fail to have their intended outcomes. In particular, they should pay attention to specific contributions that specific interventions and combinations make, so as to inform wider cost/benefit analyses that will be necessary in such scenarios.

We agree with those who have called for a more coherent response framework, facilitating clear-headed decisions between options [13], and with those who have highlighted the dangers of simplified messages that are inaccurate and lead to widespread misconceptions [17].

## Author note

This research was not supported by any grant.
